# Air pollutants associated with smoking in indoor/outdoor of waterpipe cafés in Tehran, Iran: Concentrations, affecting factors and health risk assessment

**DOI:** 10.1038/s41598-019-39684-3

**Published:** 2019-02-28

**Authors:** Mohammad Reza Masjedi, Farhad Taghizadeh, Sanaz Hamzehali, Sonia Ghaffari, Mehdi Fazlzadeh, Ahmad Jonidi Jafari, Sadegh Niazi, Ehsan Abouee Mehrizi, Masoud Moradi, Hasan Pasalari, Hossein Arfaeinia

**Affiliations:** 1 Professor of pulmonary medicine, Tobacco Control Research Center (TCRC), Iranian Anti-tobacco Association, Tehran, Iran; 2 Department of Environmental Health Engineering, School of Public Health, Iran University of Medical Sciences, Tehran, Iran; 3 Researcher, Tobacco Control Research Center (TCRC), Iranian Anti-tobacco Association, Tehran, Iran; 4 Social Determinants of Health Research Center, Ardabil University of Medical Sciences, Ardabil, Iran; 5 Department of Environmental Health Engineering, School of Public Health, Tehran University of Medical Sciences, Tehran, Iran; 6 Research Center for Environmental Health Technology, Iran University of Medical Sciences, Tehran, Iran; 7 International laboratory for air quality and health, Queensland University of Technology (QUT), Brisbane, Australia; 8 Department of Environmental Health Engineering, Faculty of Health, North Khorasan University of Medical Sciences, Bojnurd, Iran; 9 Research Center for Environmental Determinants of Health, Kermanshah University of Medical Sciences, Kermanshah, Iran; 10 Department of Environmental Health Engineering, School of Public Health, Bushehr University of Medical Sciences, Bushehr, Iran; 11 Systems Environmental Health and Energy Research Center, The Persian Gulf Biomedical Sciences Research Institute, Bushehr University of Medical Sciences, Bushehr, Iran

## Abstract

Despite increasingly growth in waterpipe smoking in Tehran, so far no study has been conducted on the air quality of the waterpipe and cigarette cafés. Thirty-six cafés were selected and the concentration of three pollutants including formaldehyde, carbon monoxide and nicotine were measured in both indoor and outdoor air of cafés two times (week-day and weekend’s session). Air sampling was performed for 180 min for each pollutant. It was observed that the concentration of pollutants inside the cafés was higher during weekend session (with a higher number of “active waterpipe heads”) compared with findings during the week-day sessions. Furthermore, the concentration of pollutants in the indoor air of the cafés was significantly higher than that of the outdoors (p < 0.05). According to path analysis, the number of “active waterpipe heads” had the maximum impact on generation of pollutants inside the cafés, followed by the type of tobacco as the second influential factor. The average of lifetime cancer risk (LTCR) resulted by formaldehyde exposure through inhalation in waterpipe (WS), cigarette (CS), waterpipe and cigarette (WCS) and none-smoking (NS) cafés in week-day and weekend sessions were estimated to be 111 × 10^−5^ and 61.2 × 10^−5^, 33.7 × 10^−5^ and 39.4 × 10^−5^, 271 × 10^−5^ and 322 × 10^−5^, and 4.80 × 10^−5^ and 5.90 × 10^−5^, respectively, which exceed the limit value by the U.S.EPA and WHO. The concentration of pollutants in the indoor air of smoking cafés in Tehran is significantly high, such that it can pose serious risks for the health of both personnel and customers. Therefore, decision makers are expected to enact applicable and strict policies so as to abate this public health risk.

## Introduction

Over the past decades, it has well been established that waterpipe tobacco smoking (WTS) causes various health threats for consumers and those are exposed to them^1^. Tobacco smoking with waterpipe, also known as Hubbly-bubbly smoking, Sheesha, Ghalyun, Narghile, Shisha, and Hookah, is common in Arab, North African, American, European, and especially Middle Eastern countries including Iran^2^. Second-hand tobacco smoke, as with its mainstream smoke, is a complex compound containing large amounts of different pollutants and chemicals, of which some compounds are known carcinogenic^3,4^. Epidemiological and experimental studies have profoundly evidenced that waterpipe smoke causes increased incidence of lung cancer, cardiovascular diseases, respiratory diseases, and other respiratory problems including bronchitis, asthma, etc.^5^. Thus, the environmental tobacco smoke (ETS) has been categorized as a human carcinogenic agent^6,7^.

As mentioned previously, various studies have indicated that waterpipe smoke contains different compounds of various harmful pollutants including particulate matters (PM_10_, PM_2.5_), carbon monoxide (CO), nitrogen oxides (NO_x_), volatile organic compounds (VOCs), volatile aldehydes (e.g. formaldehyde carcinogenic compound), poly aromatic hydrocarbons (PAHs), nicotine, phenol, furans, etc.^8–11^. According to the reports by different researchers, these pollutants are found at higher concentrations inside waterpipe cafés, as compared with the outdoor air. For instance, in a study recently conducted by Fazlzadeh *et al*., it was found that mean concentration of CO was 24.75 and 2.65 ppm in the indoor and outdoor air of the waterpipe café, respectively^10^. In addition, Zhang *et al*. reported that the concentration of PM_2.5_ particles in the air inside the waterpipe cafés (1419 μg/m^3^) was far larger than its concentration outdoors (80.5 μg/m^3^). Large amounts of these pollutants indoors consequently diminish quality of the indoor air, predisposing the people to serious risks including cardiovascular diseases, lung cancer, bronchial asthma, and other respiratory diseases^12^.

In Tehran metropolitan, there are many waterpipe cafés, which serve different tobaccos including fruit flavored, Khansar, Kashan, Hakan, etc. types. These waterpipe cafés are packed with waterpipe smokers on most days of the year, especially on holidays. Nevertheless, unfortunately no study has been conducted to measure the level of pollutants in the indoor or outdoor air of Tehran waterpipe cafés. Meanwhile, one of the main challenges encountered by policymakers and public health officers for controlling tobacco products is the lack of enough information about the quality of indoor air inside cafés, waterpipe cafés, and other similar environments, to which the consumers and personnel of the waterpipe cafés are exposed. Thus, in the present study, the concentration of formaldehyde, carbon monoxide, and nicotine was examined in the indoor and outdoor air of waterpipe cafés of Tehran. Thus, the innovation of this study is (1) investigating the quality of indoor air of Tehran cafés and comparing with the outdoor air quality, (2) investigating the concentration of formaldehyde pollutant inside waterpipe cafés for the first time in the Middle East, (3) investigating the simultaneous effect of waterpipe and cigarette cafés on air pollutants in the indoor and outdoor air, (4) comparing concentrations of CO, formaldehyde, and nicotine in the cafés that use fruit-flavored tobacco and those that serve traditional tobacco, and (5) assessing the cancer and non-cancer risks of formaldehyde in indoor air of waterpipe cafés.

## Material and Methods

### The site of study and sampling methodology

In this study, the indoor air quality of waterpipe cafés was examined in Tehran (Longitude: 35.6892°N and Latitude: 51.3890°E), capital of Iran. In Tehran, three types of waterpipe cafés are active. The first types are those in which waterpipe is served with traditional tobacco. The customers of these cafés are typically middle-aged and elderly people, who are used to consuming traditional tobaccos. These people are not interested in today’s flavored tobaccos, and thus they do not attend coffee shops where flavored tobaccos are served. The second type include the cafés in which tobaccos with fruit-flavored are used. In contrast to the first group, the customers of this group are mostly teenagers and the youth. Finally, the third type include the cafés in which both fruit-flavored and traditional tobaccos are served there. Totally, 36 cafés were selected and the concentration of three pollutants including formaldehyde, carbon monoxide, and nicotine were investigated in indoor and outdoor air from Dec 2017 to Mar 2018 (Main characteristics about smoking cafés in sample sites are provided in Table 1). Of which, fourteen sampling stations were cafés in which only waterpipe was smoked (hereafter referred to as WS), eight of them were cafés in which only cigarette was smoked (hereafter referred to as CS) and in addition, six of them were cafés in which both waterpipe and cigarette were smoked (hereafter referred to as WCS). Finally, 8 of the cafés in which no tobacco smoked such as supermarkets were chosen as the blank sample (hereafter referred to as NS). Of note, some cafés were located in the basement, while some others were situated in the ground floor. Before starting the sampling, at first the necessary explanations were given to persuade the owners and managers of the selected cafés to acquire the permission of taking samples from their indoor air. Once they were persuaded, the sampling operations were initiated. For each café, background information including the area of the properties, mode of ventilation (natural (window opening), air conditioning, water cooler), the number of doors and windows, the number of ventilators, the ventilation rates, the number of active waterpipe heads, the type of tobacco (fruit-flavored or traditional tobacco) and other information were registered using a researcher-made questionnaire. The sampling was performed during rush working hours (from 17 PM to 21 PM). The samples were taken from each sampling station twice (once on a week-day (e.g. Monday) and another time on a weekend holiday (such as Thursday or Friday) (due to the large number of active waterpipe heads). The sampling of the three selected pollutants was conducted simultaneously in the indoor and outdoor air of the waterpipe cafés.

**Table 1:** Main characteristics in smoking cafés in sample sites.

Sampling stations	Building Type^a^	Mode of ventilation^b^	Number of window and door	Number of air vent valve	Ventilation rate (m^3^/s)	Tobacco type^c^	Floor level^d^	Active waterpipe headsduring working-day session	Active waterpipe headsduring weekend session	Area of room (m^2^)
S_1_	Hookah	N and W	4	2	13.3	Flavored	Ground	5	9	6*10
S_2_	Non-smoking	N	1	1	5.84	—	Ground	—	—	3*4
S_3_	Hookah	N	5	3	16.4	Regular	Ground	6	8	8*12
S_4_	Non-smoking	N	2	1	7.12	—	Ground	—	—	4*6
S_5_	Cigarette	N	2	2	9.40	Cigarette	Basement	3	5	6*8
S_6_	Hookah	N and W	3	—	12.0	Fruit flavored	Ground	6	9	6*8
S_7_	Non-smoking	N	2	1	6.89	—	Ground	—		3*5
S_8_	Hookah and cigarette	N and W	1	2	7.80	Fruit flavored, Regular and Cigarette	Basement	11	14	8*16
S_9_	Non-smoking	N	1	1	6.23	—	Ground	—	—	3*4
S_10_	Hookah	N	3	2	13.5	Fruit flavored and regular	Ground	5	8	6*10
S_11_	Non-smoking	AC	4	8	24.0	—	Ground	—	—	12*18
S_12_	Hookah	N and W	3	4	14.0	Regular	Ground	7	11	5*7
S_13_	Hookah and cigarette	N	1	4	11.2	Fruit flavored and Cigarette	Basement	9	13	7*11
S_14_	Hookah	W	1	—	8.60	Regular	Basement	7	12	8*13
S_15_	Non-smoking	AC	1	2	7.88	—	Basement	—	—	6*10
S_16_	Hookah	N	2	3	12.4	Fruit flavored	Ground	6	8	4*6
S_17_	Hookah and cigarette	N	3	5	6.20	Fruit flavored and cigarette	Ground	7	11	10*15
S_18_	Cigarette	W	1	2	8.90	Cigarette	Ground	3	5	5*7
S_19_	Hookah	W	1	4	8.20	Fruit flavored	Basement	9	12	6*9
S_20_	Hookah	N	1	3	6.30	Regular	Basement	10	14	8*12
S_21_	Hookah and cigarette	W and N	2	6	10.5	Fruit flavored and cigarette	Ground	16	19	10*15
S_22_	Non-smoking	AC	2	5	18.0	—	Ground	—	—	8*10
S_23_	Hookah	N	2	4	7.80	Regular	Ground	5	8	6*8
S_24_	Hookah	N	2	3	8.60	Fruit flavored	Ground	7	9	7*9
S_25_	Hookah and cigarette	N and W	3	3	7.40	Fruit flavored and cigarette	Ground	13	12	8*10
S_26_	Hookah	W	1	2	8.30	Fruit flavored	Basement	8	11	10*12
S_27_	Cigarette	N	1	2	8.90	Cigarette	Ground	3	4	6*8
S_28_	Hookah	N	3	—	7.90	Fruit flavored	Ground	8	9	5*7
S_29_	Cigarette	N	1	1	10.9	Cigarette	Ground	4	7	6*8
S_30_	Non-smoking	AC	4	1	18.0	—	Ground	—	—	6*8
S_31_	Hookah	N	1	2	15.2	Regular	Basement	7	8	4*6
S_32_	Cigarette	N and W	1	3	7.50	Cigarette	Basement	5	8	8*12
S_33_	Cigarette	N	2	2	6.40	Cigarette	Ground	2	4	6*8
S_34_	Hookah and cigarette	N	3	6	8.20	Fruit flavored and cigarette	Ground	10	13	8*10
S_35_	Cigarette	N	1		9.50	Cigarette	Ground	4	6	6*8
S_36_	Cigarette	N	1	1	8.40	Cigarette	Ground	3	8	6*8

^a^Hookah, hookah and cigarette, cigarette, non-smoking.

^b^N: Natural (window opening), W: Water cooler, A: Air-Conditioning.

^c^Regular, fruit flavored.

^d^Ground floor, Basement.

### The sampling equipment and analysis of the pollutants

All of the equipment used for sucking the air, sampling, real-time measurement, analytical devices, etc. were calibrated before the experimentation. Note that the sampling equipment was devised both inside and outside the waterpipe café in respiratory region (placed 100–120 cm above the ground level). The ambient samples were taken from a distance of lower than 50 m off the waterpipe café and 5 m off the main street, simultaneously with the indoor sampling. In fact, the sampling from the outdoor air was done in the streets around the café building both for cafés located in the ground-floor and for those situated in the basement. In those streets, vehicle’s transportation, etc. occurred. Note that the outdoor air sample for the basement cafés didn’t take place from a closed space. The details of the sampling for the three target pollutants including, formaldehyde, carbon monoxide, and nicotine) are provided further.

#### Formaldehyde

In the case of formaldehyde, two sampling setups were run simultaneously; one inside and the other outside the waterpipe cafés. To measure the formaldehyde concentration, Chromotropic acid method was employed^13^. For this purpose, a personal sampling pump (SKC, USA) was used with a flow rate of 1 L/min. Air suction into the set up was performed for three hours continuously (the volume of each sample taken was 180 L). For effective absorption of formaldehyde, two impingers were placed serially, and 25 ml of sodium bisulfite (1%) was injected into each of them. To prevent entrance and absorption of particulate matters into the impingers, PTFE filter was placed in their input aperture. Thereafter, the samples were transferred to low-density polyethylene pipes and then transferred to laboratory to analyze the formaldehyde concentration.

In the laboratory, 5 ml of each sample was poured into 25-ml flasks. 0.1 ml of Chromotropic acid (1%) was added as a reagent to the solution, with the mixture being stirred slowly for 2 min. Next, 6 ml of sulfuric acid was added to the samples and stirred leniently. The obtained solution was placed inside a water bath at 95 °C for 10 min, and then cooled down at laboratory temperature. Eventually, to measure the concentration of formaldehyde in each sample, spectrophotometer device (V/Vis Spectrophotometer, Hach-DR 5000, USA) at the wavelength of 580 nm was used; calibration curve had been prepared in accordance with Beer-Lambert law. For accurate determination of the pollutant values, the blank samples were also prepared and read alongside the main samples. The limit of detection (LOD) and limit of quantification (LOQ) for formaldehyde were calculated 0.027 and 0.059 μg/m^3^, respectively.

#### Carbon monoxide (CO)

To measure the concentration of carbon monoxide, portable CO detector (CO-mètre - CO 50, Kimo Instruments,Inc. Canada) was used. In this case, measurement was also performed concurrently for the indoor and outdoor air of the waterpipe café. Measurement of CO concentration was conducted continuously for 180 min, and every four minutes, the concentration observed in the detector screen was noted down. After measurement completion, the average value of 36 notes was reported as the concentration of CO in each sampling station. The measurement range of this detector was 0–500 ppm, whose accuracy for 0–100 ppm and 100–500 ppm concentrations was ±3 ppm and ±3% of reading, respectively.

#### Nicotine

NIOSH 2551 method was employed to measure the nicotine in indoor air of sampled cafés. In this method, at first, two heads of the XAD-4 sampling tubes were broken and according to the side of the arrow in the tube surface, it was connected to the flexible tubes of the personal sampling pump. Thereafter, air suction into these tubes was continued for 180 min at a flow rate of 0.5 L/min (the size of each sample taken was 90 L). After the sampling, XAD-4 tubes were detached off the pump and immediately its two sides were plugged by plastic caps specifically designed for it. Thereafter, the tubes were transferred to the laboratory away from the sunlight and beside dry ice. In the laboratory, the absorbent in the front and rear parts (separated by glass wool plug) of the sampling tube was poured into separate vials and 1 mL of ethyl acetate (as the desorbing solution) was added to each of them. Next, to extract nicotine from the absorbent, the vials containing the sample were placed inside the ultrasonic bath for 30 min. Eventually, to determine the amount of nicotine in the samples, 1 µL of the solution of each vial was taken and injected into gas chromatography (GC) device equipped with FID detector (Agilent 7890 A) (according to 2551 NIOSH method). Around 10% of the samples injected into the device consisted of environmental blank and experimental samples. LOD was calculated in the target ion as the analyte concentrations providing a S/N value equal to 3, as determined by the Agilent MSD proprietary software (“Chemstation”). LOD of 0.41 μg/m^3^ was found for nicotine. LOQ was calculated by adding to the mean of blank sample a value of 10 times its standard deviation; it was 1.03 μg/m^3^ for formaldehyde.

### Health risk assessment

Given that carcinogenic effects of carbon monoxide and nicotine to human have not yet been proved, and on the other side formaldehyde is established as a probable human carcinogen (group B1)^14^, in this study mere formaldehyde exposure was considered for assessing human health risk.

To assess the risk to the human health upon exposure to formaldehyde, their inhalation lifetime cancer risk (LTCR) and hazard quotient (HQ) was estimated. The LTCR and HQ was calculated as follows:

1 $${\rm{E}}={\rm{C}}\times {\rm{IRa}}\times {\rm{EDa}}/{\rm{BWa}}$$

2 $${{\rm{E}}}_{{\rm{Y}}}={\rm{C}}\times {\rm{IRa}}\times {\rm{EDa}}\times ({\rm{D}}/7)\times ({\rm{Wk}}/{\rm{52}})/{\rm{BWa}}$$

3 $${{\rm{E}}}_{{\rm{L}}}={\rm{E}}\times ({\rm{D}}/{\rm{7}})\times ({\rm{Wk}}/{\rm{52}})\times ({\rm{YE}}/{\rm{YL}})$$

4 $${\rm{LTCR}}={{\rm{E}}}_{{\rm{L}}}({\rm{mg}}/{\rm{kg}}{\rm{.d}})\times {\rm{SF}}\,({\rm{mg}}/{\rm{kg}}{\rm{.d}})$$

5 $${\rm{HQ}}={{\rm{E}}}_{{\rm{Y}}}/{\rm{RfD}}$$

where, E: daily exposure (mg/kg.d), E_Y_: yearly average daily dose received (mg/kg.d), E_L_: effective life time exposure (mg/kg.d), HQ: hazard quotient, and RfD; reference dose (mg/kg.d)^15,16^. Risk parameters used for calculating LTCR and HQ for formaldehyde are shown in Table 2^15^.

**Table 2:** Risk parameters used for calculating LTCR and HQ for formaldehyde^15^.

	Value		Unit
	Indoor	outdoor	
Concentration of the pollutant (C)	—	—	mg/m^3^
Inhalation rate, adult (IRa)	0. 830	0.830	m^3^/hr
Exposure Duration, adult (EDa)	8.00	24.0	hr/d
Body Weight, adult (Bwa)	70.0	70.0	Kg
Days per Week Exposure (D)	6.00	7.00	d
Weeks of Exposure (WK)	48.0	52.0	week
Years of Exposure (YE)	30.0	70.0	Y
Years in Lifetime (YL)	70.0	70.0	Y
Slope factor or carcinogenic potency slope (SF)	formaldehyde = 0.046		(mg/kg.d)
Reference concentration (RfC)	9.8 × 10^−3^ ^41^		µg/m^3^
Reference dose (RfD)	2.78 × 10^−3^		mg/kg-day

RfD = RfC (inhalation reference concentration mg/m^3^) × Assumed inhalation rate (m^3^/day) × 1/BW (kg)^19,42^.

According to the World Health Organization (WHO), LTCR values considered as “an acceptable limit for humans” are proposed to range from 1.00 × 10^−5^ to 1.00 × 10^−6^, but LTCR values less than 1.00 × 10^−6^ are recommended by U.S.EPA^17,18^. Moreover, HQ values higher than 1.00 considered as “ high non-carcinogenic risk”, but HQ values less than 1.00 is recommended value by Agency for Toxic Substances and Disease Registry (ATSDR)^19^.

### Statistical analyses

The data were analyzed by Microsoft Excel. Data normality was evaluated by Kolmogorov-Smirnov test (K-S test). The significance of the difference of the pollutants’ concentration between the indoor and outdoor air was examined by two sample t-test. Furthermore, significance of the difference of the pollutants’ concentration in the indoor air of the waterpipe cafés and the indoor air of the premises in which tobacco was not smoked was also investigated by two sample t-test. To study the linear relationship between the values measured for the pollutants in the indoor and outdoor environment of the waterpipe cafés, Pearson correlation test was utilized. The level of significance of the tests was considered as 0.05 and 0.01 (confidence level of 95 and 99%). Finally, path analysis through Amos 21 was conducted to determine the factors that have a significant effect on the concentration of pollutants inside the waterpipe cafés.

## Results and Discussion

### Pollutants concentrations in indoor/outdoor of waterpipe cafés

#### Carbon monoxide (CO)

The concentration obtained in this study for the carbon monoxide (CO), formaldehyde, and nicotine for the indoor and outdoor air of the cafés is provided in Table 3. Comparatively, the concentration of pollutants in indoor air of WCS cafés were the highest followed by WS, CS and NS. Specifically, the mean ± SD of CO concentration for WS, CS, and WCS has been 8110 ± 3540, 5560 ± 22404, and 11300 ± 4840 µg/m^3^ during the week-day session, while the values have been 8660 ± 3810, 6590 ± 2110, and 13600 ± 5480 µg/m^3^ during the weekend session. No value was observed for this pollutant at non-smoking sites. The mean ± SD of the CO concentration in the outdoor air during the week-day and weekend sessions were 1990 ± 1280 and 631 ± 314 µg/m^3^, respectively. Considering CO concentration in the indoor air of the cafés, it should be noted that the concentration of this pollutant had not exceeded the guideline presented by WHO (32000 µg/m^3^ for 1 h) in any of the sample cafés. The values observed for CO in this study have been lower than the value reported for the indoor air of waterpipe cafés in Ardabil^10^. Nevertheless, two scenarios can be considered for exposure to CO inside the tobacco cafés: occupational exposure and general population exposure. Regarding occupational exposure, the workers and personnel of any of cafés are exposed to high amounts of occupational OEL-TWA proposed by Iranian health ministry (28000 µg/m^3^ for 44 working hours in the week). In the second scenario, the pollutant affects the customers and general people who come to these cafés for activities other than waterpipe smoking (e.g. drinking tea, etc.), and normally spend around one hour of their routine day in these cafés. In comparison to the guidelines proposed by WHO (32000 µg/m^3^ for 1 hr), none of the customers are at risk of exposure to high CO concentrations. Long-term exposure to CO plays a significant role in aggravating many cardiovascular and respiratory diseases including cardiomyopathy, atherosclerosis, chronic obstructive pulmonary disease, asthma, and respiratory infections^20^. CO poisoning after waterpipe smoking has been reported in some case reports where the level of carboxyhemoglobin present in the consumer’s blood had gone beyond 34% of the normal limit^21,22^.

**Table 3:** Descriptive statistics for indoor and outdoor CO, formaldehyde, and nicotine concentrations in the waterpipe café of Tehran.

Smoking building type (N)^a^			CO		Formaldehyde		nicotine	
			Mean ± SD	Min–max	Mean ± SD	Min–max	Mean ± SD	Min–max
Waterpipe (14)	working-day session	Indoor	8110 ± 3540	5040–13100	549 ± 290	121–931	5.06 ± 2.40	1.40–10.3
		Outdoor	2090 ± 981	799–3780	14.0 ± 8.00	5.00–28.0	ND	ND
	Weekend Session	Indoor	8660 ± 3810	1610–16300	618 ± 287	181–1110	6.91 ± 2.47	3.70–12.6
		Outdoor	501 ± 321	111–921	12.0 ± 7.00	4.00–21.0	ND	ND
Cigarette (8)	working-day session	Indoor	5560 ± 2240	4710–6640	151 ± 29.0	121–189	2.32 ± 0.850	1.39–3.41
		Outdoor	1350 ± 601	899–1830	10.6 ± 6.51	5.81–17.6	ND	ND
	Weekend Session	Indoor	6590 ± 2110	5610–7670	184 ± 34.0	131–211	4.02 ± 0.890	3.00–5.10
		Outdoor	811 ± 451	401–1260	9.50 ± 7.30	4.31–19.7	ND	ND
Waterpipe and cigarette (6)	working-day session	Indoor	11300 ± 4840	7801–15400	1340 ± 211	107–165	13.1 ± 2.68	9.80–15.6
		Outdoor	2120 ± 1710	701–5380	119 ± 8.00	4.00–21.0	ND	ND
	Weekend Session	Indoor	13600 ± 5480	8710–19800	1620 ± 286	124–199	16.8 ± 3.98	12.5–21.0
		Outdoor	641 ± 369	299–1030	16.0 ± 12.0	4.00–28.0	ND	ND
Non-smoking (8)	working-day session	Indoor	ND	ND	46.0 ± 25.0	34.0–55.0	ND	ND
		Outdoor	1760 ± 601	919–2860	15.0 ± 12.0	4.00–31.0	ND	ND
	Weekend Session	Indoor	ND	ND	57.0 ± 36.0	22.0–93.0	ND	ND
		Outdoor	599 ± 419	201–1260	19.0 ± 11.0	9.00–27.0	ND	ND

^a^No. of Samples.

#### Formaldehyde

The mean ± SD of formaldehyde concentration in WS, CS, WCS cafés as well as non-smoking sites were found to be 549 ± 290, 151 ± 29.0, 1340 ± 211, and 46.0 ± 25.0 µg/m^3^ in week-day session, while for the weekend, the values were 618 ± 287, 184 ± 34.0, 1620 ± 286, and 57.0 ± 36.0 µg/m^3^. Finally, the mean ± SD of the formaldehyde concentration in the outdoor air during the week-day and weekend sessions was 12.8 ± 8.76 and 14.9 ± 8.27 µg/m^3^, respectively. As seen, the concentration of this pollutant in the indoor air of HC and CS cafés was quite high, such that its mean concentration in the indoor air of the cafés was far higher than the exposure limits presented by Iranian and international organizations. This suggests unsafe and dangerous conditions for the personnel and customers who spend a short time (longer than 15 min) inside these cafés, even they have no smoking activity. The concentration of this pollutant in non-smoking premises did not exceed the allowable limits. Since no study had been conducted for investigating the concentration of formaldehyde in the indoor air of waterpipe cafés, the values found in this study were only compared with the standards and guidelines proposed by different organizations, and we were not able to compare the values with other studies. In this research, it was also observed that formaldehyde concentration in the ambient air had not exceeded the guideline presented by WHO (100 µg/m^3^). Various other studies have also reported low values for this pollutant in the atmosphere of cities. An ambient air concentration of lower than 10ppb and around 12ppb was observed for the atmosphere of São Paulo, Brazil^23^ and Rome, Italy^24^.

#### Nicotine

During the week -day session, the mean ± SD concentration of nicotine in WS, CS, and WCS cafés has been 5.06 ± 2.40, 2.32 ± 0.850, and 13.1 ± 2.60 µg/m^3^, while for the weekend session, the values have been 6.91 ± 2.47, 4.02 ± 0.890, and 16.8 ± 3.98 µg/m^3^. Again, no value was observed for this pollutant at non-smoking sites (Table 3). The mean values of nicotine concentration observed in this study were much lower for the indoor air of WS, CS, and WCS (5.98 ± 2.42, 3.17 ± 0.89, and 14.9 ± 3.21 µg/m^3^ respectively) compared with the occupational standards stated by occupational safety and health administration (OSHA) (500 µg/m^3^). Nevertheless, these standard values are employed for healthy workers and personnel, and may not be applicable to sensitive individuals including children, the elderly, and patients. The concentrations of nicotine observed in this study have been larger than the value reported for Hookah Bars in New York (4.2 µg/m^3^)^25^ and Toronto (3 µg/m^3^)^26^. In addition to its potential for addiction and as a gateway to using other tobacco products, nicotine has very harmful health complications such as negative effects on the nervous system^27^, disorder in the formation, survival, and differentiation of brain cells^28^, low infant weight during childbirth after exposure during pregnancy^29^, diminished hearing^30^, dental decay^31^, and hyperactivity^32^. Probably, the most important point was that across all of the cafés in which tobacco was smoked, measurable concentration of nicotine was observed. This suggests that there is an immediate need for formal assessment in order to see whether these observations are compatible with the Iranian national law regarding prohibition of tobacco use in public places. The findings increase serious concerns about potential untoward health effects among the customers and personnel of the smoking cafés.

Considering pollutant concentration in the outdoor air of the cafés, the concentration values have been significantly lower compared with the indoor environment (P value < 0.05). Note that no value was observed for nicotine in the outdoor air across any of the sampling sites. Further, no significant relationship was observed between the concentration of the pollutants in the indoor and outdoor environment of the cafés (R = −0.19, Pvalue = 0.11).

### Indoor/outdoor (I/O) ratios

The findings of the indoor/outdoor (I/O) ratios for target pollutant are indicated in Table 4. It was observed that, the mean (I/O) ratios of formaldehyde concentration in WS, CS, and WCS were 37.7, 20.7, and 156, respectively. This value for CO in WS, CS, and WCS were 5.75, 6.03, and 10.1, respectively. Due to the lack of data on the concentration of nicotine in the outside air, it is not possible to calculate the I/O ration for this pollutant. Indoor/outdoor (I/O) ratio is a criterion for assessing the difference between indoor pollutant levels and the corresponding outdoor concentrations or as an index for the strength of sources in indoor of buildings. Indoor pollutants concentration are influenced by infiltration of outdoor pollutants into the buildings and indoor sources^33^. I/O ratio can vary largely because of a large number of factors such as building design, locations and various indoor activities^34^. This ratio has been calculated to compare the dynamics between indoor and outdoor concentration of air pollutants in WS, CS, WCS and NS. In terms of health, ratio below 1 is clearly preferable, showing that the cafés provides some technique of reduction to outside pollutants exposure^35,36^. As mentioned earlier, the I/O ratios for target pollutant in these cafés (WS, CS and WCS) were all calculated to be significantly higher than 1, which implies the presence of an important source (smoking) for indoor pollutants releasing. But the I/O ratio for pollutants in NS was found to be close to or below 1, indicating that operational and/or fabric environment of these cafés do not perform an effective function relating to reduction of indoor air pollutant concentration. Based on the I/O ratio, since the concentration of the target pollutants in this study was far lower in the outdoor air as compared with the indoor air, diffusion of atmospheric pollutants to indoor environments in our study is negligible. However, the waterpipe and cigarette cafés can be considered an important source for emission of these pollutants across the atmospheric air on a local scale.

**Table 4:** Indoor-to-outdoor ratios (I/O) for CO, formaldehyde, and nicotine in the waterpipe cafés of Tehran.

Café type	I/O Ratio		
	CO	Formaldehyde	nicotine
HS	5.75	34.7	NC^*^
CS	6.03	20.7	NC
HCS	10.1	156	NC
NS	NC	NC	NC

*Not-calculable.

### Factors affecting pollutants concentrations

**Figure 1:**
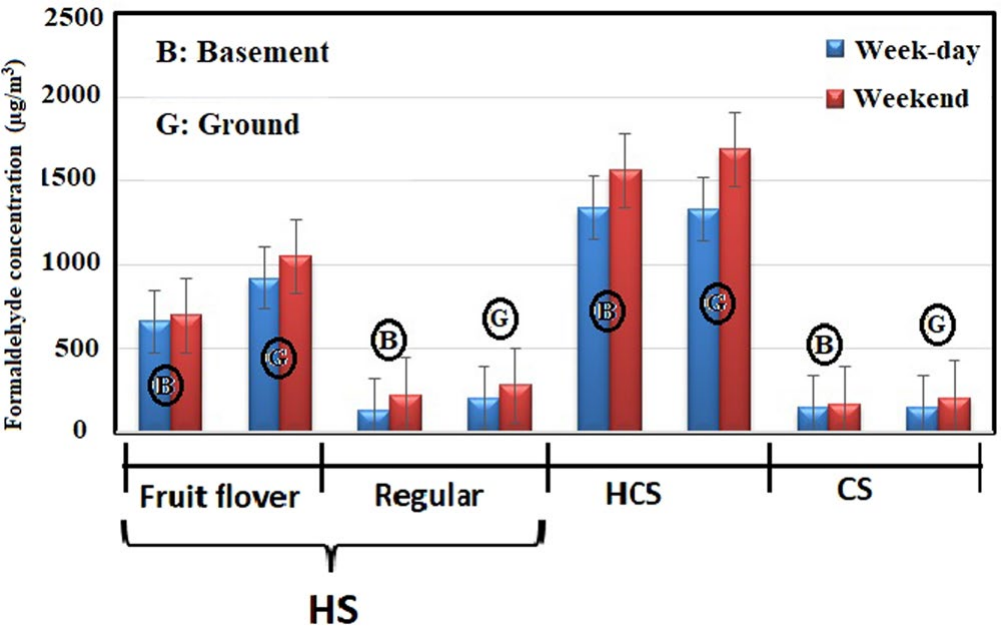
Mean concentration of formaldehyde in waterpipe cafés based on number of active waterpipe heads, tobacco type, and the floor level.

**Figure 2:**
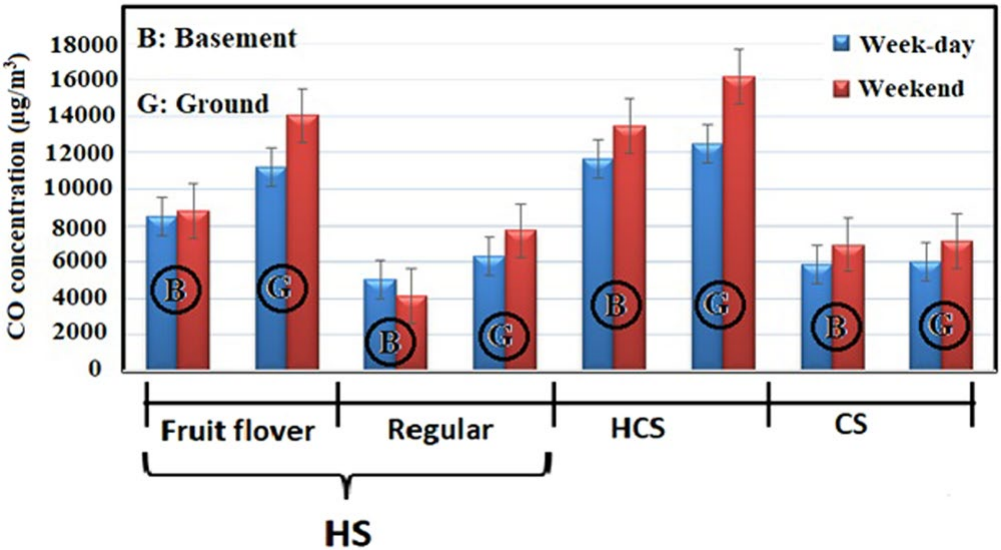
Mean concentration of CO in waterpipe cafés based on number of active waterpipe heads, tobacco type, and the floor level.

**Figure 3:**
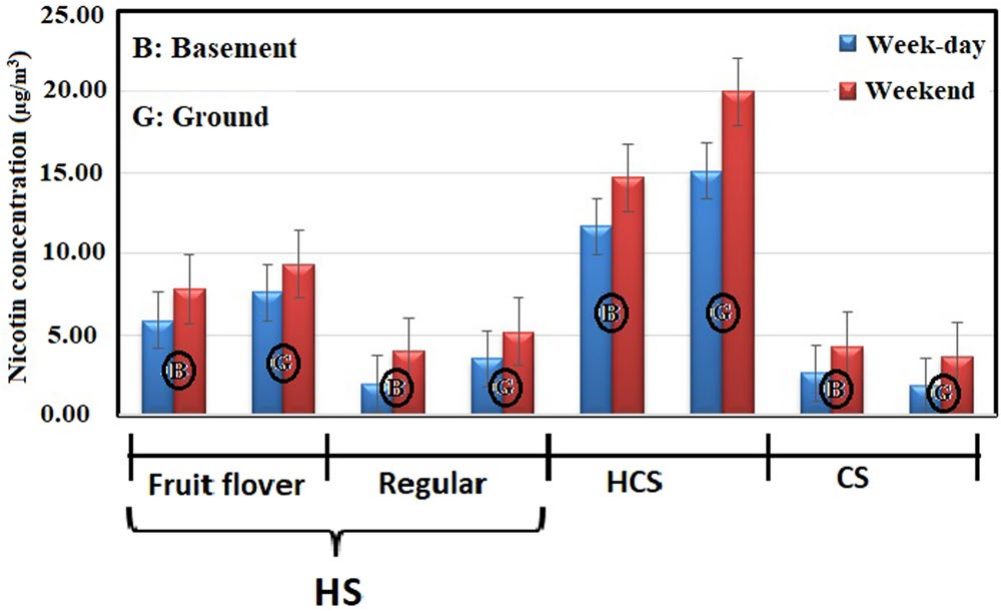
Mean concentration of nicotine in waterpipe cafés based on number of active waterpipe heads, tobacco type, and the floor level.

Amongst the various influential factors on indoor air quality, number of “active waterpipe heads”, the tobacco type, and the location floor of café were studied. Number of “active waterpipe heads” is discussed in section 3.2 in detail. Given the type of tobacco, out of the 14 waterpipe cafés, fruit-flavored and traditional tobaccos were smoked in eight and six of them, respectively. The mean concentration of the pollutants in the waterpipe cafés with fruit-flavored tobacco was significantly higher than the traditional counterpart. The mean concentration of formaldehyde, carbon monoxide (CO), and nicotine in the waterpipe cafés with fruit-flavored tobacco was 856 ± 267, 1190 ± 2150, and 7.20 ± 0.678 µg/m^3^, respectively. On the other hand, the values in the waterpipe cafés with traditional tobacco have been 176 ± 54.6, 6120 ± 887, and 3.79 ± 0.343 µg/m^3^, respectively. The higher concentration of CO in fruit-flavored cafés might be interpreted through the time required to smoke waterpipe with different type of tobacco. Waterpipes containing flavored tobacco last at least 4 times longer to smoke than the traditional one. This may be due to the soft and tasty smoke of flavored tobacco as well as the tendency of youth customers to spend more time on smoking of this type of waterpipe^10^. Moreover, fruit- flavored tobaccos contain large amounts of organic chemical compounds, aroma, essences and flavoring additives, which are added to this type of tobaccos during the manufacturing process^37^. High formaldehyde concentration in cafes offering fruit-flavored tobacco can be attributed to these chemical compounds. Furthermore, the concentration of pollutants inside the cafés located in the basement was significantly higher than those situated in the ground floor (Figs 1–3).

Through path- analysis, the effect of the properties and different factors in emission of pollutants inside the studied cafés was evaluated. With this analysis, modulus standardized effect sizes (MSES) for the number of active waterpipe heads, type of tobacco, and floor level of café were estimated to be 0.46, 0.34, and 0.26, respectively. Accordingly, the next influential factor has been the type of tobacco used in the cafés. According to the path-analysis, among the variables that may influence emission of pollutants inside the cafés, “the number of active waterpipe heads” and “tobacco type” have had the maximum impact. It was observed that the concentration of the pollutants in the indoor environment of the cafés was higher during the weekend session (with a larger number of active waterpipe heads), as compared with week-day sessions. Compared with cigarette smoke, different types and values of pollutants are produced in response to waterpipe smoking. High exposure to high molecular weight PAHs and benzene, but lower exposure to propylene oxide, acrolein, acrylonitrile, butadiene-1,3, nitrosamines, ethylene oxide, and low molecular weight PAHs have been reported in tobacco cafés as compared with cigarette cafés^38^. Different rates of pollutants production by different tobacco products have been observed in previous studies^39,40^. In the present study, the tobacco type with MSES (0.34) has been the second influential factor in the emission of the selected pollutants in this study. In this regard, in WSs in which fruit-flavored tobacco was smoked, the extent of pollutants releasing was significantly higher than in WSs in which traditional tobacco was used.

Similar results have been reported by previous studies in generation of CO and benzene, toluene, ethylbenzene, and Xylene (BTEX)^10,16^. According to path-analysis, it was also observed that the concentration of the pollutants was significantly higher in the cafés located in the basement, as compared with those situated in the ground floor. Basements are usually confined places, which have walls without any holes and very limited natural ventilation. Since ventilation is an influential factor in refreshing the air inside premises and cafés, the cafés located in the basement suffered from higher pollutant levels, as expected. According to findings of this study, it can be suggested that the health ministry prohibit development of cafés in underground floors.

### Health risk assessment

The mean LTCR and HQ calculated for formaldehyde in indoor/outdoor air of waterpipe cafes are shown in Tables 5, 6, respectively. As can be seen in Table 5, the mean of LTCRs for formaldehyde in indoor air of WS, CS, WCS, and NS in week-day and weekend sessions were calculated were calculated as follow 111 × 10^−5^ and 61.2 × 10^−5^, 33.7 × 10^−5^ and 39.4 × 10^−5^, and 270.2 × 10^−5^ and 322 × 10^−5^, respectively, which exceed the limit value by the US EPA and WHO. Moreover, the mean of HQ for formaldehyde in indoor air of WS, CS, and WCS in week-day and weekend sessions were calculated to be 8.6 and 9.9, 2.6 and 3.1, 21.3 and 25.4, respectively (Table 6), which exceed the limit value by the ATSDR (3). As can be seen in the table, the cancer and non-cancer risks was high for all smoking cafes. But the risks was higher for WS and CS cafes, showing the high health risk of formaldehyde in these cafes. Therefore, it is suggested that the relevant organizations design and implement programs to train and raise the public awareness about the health risks caused by smoking for individuals exposed to tobacco smoke (both smokers and non-smokers). Therefore, with increased awareness of these individuals, it is possible to decrease the presence of these individuals in smoking cafés. The results also indicate that the risk of cancer in week-day sessions is higher than the weekend sessions. The mean of LTCRs for formaldehyde in outdoor air of WS, CS, WCS and NS in week-day and weekend sessions were calculated to be 15.3 × 10^−5^ and 15.5 × 10^−5^, 33.7 × 10^−5^ and 39.4 × 10^−5^, 18.5 × 10^−5^and 17.5 × 10^−5^, and 10.1 × 10^−5^ and 9.90 × 10^−5^, respectively, which this values exceed the limit value by the U.S.EPA and WHO. Also, the mean of HQ for formaldehyde in outdoor air of WS, CS, WCS and NS in week-day and weekend sessions were calculated to be 1.2 and 1.2, 0.8 and 0.7, 1.4 and 1.4, and 0.8 and 0.8, respectively, which this values in some cases exceed the limit value by the ATSDR. As the results show, the level of risk in both indoor and outdoor air is higher than the permitted levels. Moreover, the risk level in the indoor air of cafés was significantly higher than that of the outdoors. The explanatory reason is that the higher concentrations of formaldehyde are emitted from various sources in the interior of the cafés. The results also indicate that the risk level is heavily influenced by waterpipes and cigarette, such that it’s mean in HC and CS cafés was far higher than places where there is no smoker (NS). Moreover, in the places with no tobacco smoking (NS), the cancer and non-cancer risk levels of formaldehyde for outdoor air is higher than indoors. This evidently demonstrates that smoking is considered as a main source of formaldehyde in cafes, contributing to increased cancer risk in exposed people. People with a wide age range were present in these cafés. However, active smokers were generally young boys and girls who spent their leisure time with their friends and waterpipe smoke. In some cases, it was observed that children and women were also present in these cafés. Therefore, raising the awareness of women and notification about consumption of tobacco products is essential. It is also suggested that kindergartens and children schools consider some training about the harms of waterpipe and tobacco to children and students.

**Table 5:** Cancer risk (LTCR) related to formaldehyde concentrations in indoor and outdoor air of waterpipe cafés.

Cafés			Average	Min	Max	I/O ratio	
						Week-day	Weekend
Waterpipe	Indoor	Week-day	111 × 10^−5^	25.1 × 10^−5^	194 × 10^−5^	7.15	3.94
		Weekend	61.2 × 10^−5^	36.6 × 10^−5^	232 × 10^−5^		
	Outdoor	Week-day	15.3 × 10^−5^	—	39.6 × 10^−5^		
		Weekend	15.5 × 10^−5^	—	30.4 × 10^−5^		
Cigarette	Indoor	Week-day	33.7 × 10^−5^	240 × 10^−5^	47.9 × 10^−5^	3.09	4.52
		Weekend	39.4 × 10^−5^	27.4 × 10^−5^	50.7 × 10^−5^		
	Outdoor	Week-day	10.9 × 10^−5^	—	24.4 × 10^−5^		
		Weekend	8.70 × 10^−5^	—	27.3 × 10^−5^		
Waterpipe and cigarette	Indoor	Week-day	271 × 10^−5^	224 × 10^−5^	345 × 10^−5^	14.6	18.4
		Weekend	322 × 10^−5^	249 × 10^−5^	416 × 10^−5^		
	Outdoor	Week-day	18.5 × 10^−5^	5.10 × 10^−5^	291 × 10^−5^		
		Weekend	17.5 × 10^−5^	—	39.2 × 10^−5^		
Non-smoking	Indoor	Week-day	4.80 × 10^−5^	—	11.5 × 10^−5^	0.470	0.590
		Weekend	5.90 × 10^−5^	—	19.4 × 10^−5^		
	Outdoor	Week-day	10.1 × 10^−5^	—	43.4 × 10^−5^		
		Weekend	9.90 × 10^−5^	—	37.0 × 10^−5^

**Table 6:** Hazard quotient (HQ) related to formaldehyde concentrations in indoor and outdoor air of waterpipe cafés.

Cafés			Average	Min	Max	I/O ratio	
						Week-day	Weekend
Waterpipe	Indoor	Week-day	8.60	1.98	15.3	7.10	8.20
		Weekend	9.90	2.80	18.3		
	Outdoor	Week-day	1.20	—	3.10		
		Weekend	1.20	—	2.40		
Cigarette	Indoor	Week-day	2.60	1.90	3.70	3.20	4.40
		Weekend	3.10	2.10	4.00		
	Outdoor	Week-day	0.800	—	1.90		
		Weekend	0.70	—	2.10		
Waterpipe and cigarette	Indoor	Week-day	21.3	17.6	27.2	15.2	18.1
		Weekend	25.4	19.6	32.8		
	Outdoor	Week-day	1.40	0.400	2.30		
		Weekend	1.40	—	3.10		
Non-smoking	Indoor	Week-day	0.300	—	0.900	0.370	0.600
		Weekend	0.500	—	1.50		
	Outdoor	Week-day	0.800	—	3.40		
		Weekend	0.800	—	2.90		

### Limitation of the study

The present study has also some limitations, which should be considered in applying the results broadly to other tobacco cafés in other parts of the world. The first limitation is the small sample size and not selecting them randomly. In addition, the sampling time in this study was short (at most for 3 hr), and no long-term sampling (e.g. 24 hr) was conducted. A three-hour sampling cannot respond to this question “up to when after waterpipe smoking or cigarette smoking the concentration of pollutants in the indoor air remains at a dangerous level?”. In addition, the pattern of smoking is different among cigarette and waterpipe smokers, i.e. Waterpipe smoker’s smoke for a longer time but with a lower frequency. However, cigarette smokers smoke higher frequency but within a shorter time. Indeed, it is ambiguous how this difference in smoking patterns affects the accumulation and dissipation of pollutants inside the cafés. Furthermore, the pollutants assessed in this study were limited to, formaldehyde, CO, and nicotine, while the dangerous and toxic pollutants in tobacco smoke including PAHs, acrolein, propylene oxide, acrylonitrile, butadiene-1,3, nitrosamines, and ethylene oxide were not studied due to unavailability of sampling and analytical equipment and shortage of research budget. Accordingly, they can be interesting ideas for further studies.

## Conclusion

This is the first research to indicate that many waterpipe and cigarette cafés in Tehran serve various tobacco-based products. These cafés contain high levels of indoor air pollutants (formaldehyde, CO, and nicotine), which can cause a serious risk to the customers, personnel and general people. This work also presents primary data on the concentration of formaldehyde in cafés in Tehran. It was also observed that the concentration of the pollutants in the indoor environment of the cafés was higher during the weekend session (with a larger number of active waterpipe heads), as compared with week-day sessions. Moreover, significantly higher levels of indoor air pollutants (formaldehyde, CO and nicotine) was observed in the waterpipe cafés serving fruit flavored tobacco, as compared with those served regular tobacco. It was also found that the levels of the pollutants were considerably higher in the cafés located in the basement, as compared with those situated in the ground floor. Finally, the mean LTCRs for formaldehyde in outdoor air of waterpipe cafes, cigarette cafes, waterpipe and cigarette cafes and nonsmoker cafes in working day and weekend were calculated 15.3 × 10^−5^ and 15.5 × 10^−5^, 33.7 × 10^−5^ and 39.4 × 10^−5^, 18.5 × 10^−5^and 17.5 × 10^−5^, and 10.1 × 10^−5^ and 9.9 × 10^−5^, respectively, which this values exceed the limit value by the US EPA and WHO. Thus, it is necessary that better and more researches and monitoring be carried out on waterpipe and cigarette cafés and suitable tobacco control policies be arranged for this public health threat. The findings reported in this study can provide important information to inform the policy of tobacco products for the authorities of public health so that they can consider them for arranging the regulations of prohibiting tobacco use across cafés in Tehran.
